# Specific food intake, fat and fiber intake, and behavioral correlates of BMI among overweight and obese members of a managed care organization

**DOI:** 10.1186/1479-5868-3-42

**Published:** 2006-11-26

**Authors:** Jennifer A Linde, Jennifer Utter, Robert W Jeffery, Nancy E Sherwood, Nicolaas P Pronk, Raymond G Boyle

**Affiliations:** 1 Division of Epidemiology and Community Health, School of Public Health, University of Minnesota, Minneapolis, MN, USA; 2 School of Population Health, University of Auckland, New Zealand; 3 HealthPartners Research Foundation, Minneapolis, MN, USA

## Abstract

**Background:**

The study examined correlates of body mass index (BMI) in overweight and obese members of a managed care organization seeking treatment for obesity. It assessed intake of specific foods, dietary fat or fiber, and behaviors attempted to control weight.

**Methods:**

Participants were 508 men and 1293 women who were > 18 years and had a self-reported BMI > 27.0. This paper reports analyses of baseline and 24-month follow-up data from a randomized weight-loss trial. Cross-sectional and prospective relationships between BMI and behaviors were examined with regression analyses controlling for age and education.

**Results:**

At baseline, hamburger and beef consumption were associated with higher BMI for men; for women, hamburger, fried chicken, hot dog, bacon or sausage, egg, French fry, and overall fat consumption were associated with higher BMI, while eating high fiber cereal, fruit, and overall fiber intake were associated with lower BMI. Virtually all forms of weight control behavior were reported more often in heavier people. Subscribing to exercise magazines, however, was associated with lower BMI. Decreased fat intake and increased fruit/vegetable/fiber intake over the course of the study were associated with reductions in BMI at 24 months.

**Conclusion:**

The same behaviors that differentiate individuals with different body weight in the general population also differentiate between individuals of different body weights at the high end of the weight distribution. Educational efforts aimed at preventing weight gain and reducing obesity might benefit from focusing on specific foods known to be associated empirically with body weight and weight change over time.

## Background

Recent dramatic increases in the prevalence of obesity around the world [1-4] have provided an opportunity for renewed discussion of how best to advise the general public about behaviors that may be helpful in reducing body weight or preventing weight gain. Although opinions on this topic vary widely, two prominent source of information are the *Dietary Guidelines for Americans*, commissioned by the United States Departments of Agriculture and Health and Human Services [5] and the report from the World Health Organization (WHO) entitled *Global Strategy on Diet, Physical Activity and Health *[6]. These guidelines tend to be expressed in terms of nutritional attributes. For example, both sets of guidelines recommend choosing a diet that is low in saturated fat, cholesterol, sodium and sugar, moderate in total fat and high in fruit and vegetable intake. While these guidelines are widely accepted by the nutritional community, arguably they fall short of being optimal for promoting behavior change because their translation into understandable behavioral directives requires knowledge of the nutritional composition of foods that many people are not likely to possess.

Lack of specificity in public health recommendations about weight control may be explained in part by decades of dietary research that does not address behavioral specificity directly. A considerable body of research demonstrates that dietary fat intake is associated with weight and weight change [7-13]. However, information that is available on the associations of food choices or other behaviors and weight typically has been collected in general population samples [7,13], by considering macronutrient intake or general food categories rather than more specific foods [8-10,12] or in small samples with restricted assessment of specific foods [11], so that the relevance of this information in terms of behavioral specificity may be limited.

In order to better identify specific behavioral recommendations for weight control among overweight and obese adults, the goal of the current study was to examine specific food choice and other behavioral correlates of body mass index (BMI) in overweight and obese adults seeking treatment for weight loss. Frequency of intake of a number of specific foods, overall fat and fiber intake, and weight control behaviors (formal and informal) are assessed. It is believed that the size of this sample of overweight and obese treatment seekers provides a particularly unique opportunity to examine whether behaviors that are associated with differences in body weight in the general population are also associated with body weight differences in the clinically obese, treatment-seeking range.

## Methods

Data for the present study were collected as part of the assessment for a 2-year weight-loss intervention trial, Weigh-To-Be. The purpose of the overall study was to determine the effectiveness of two low-intensity weight control programs that use mail or telephone contacts to deliver weight-loss services in comparison to usual care [14,15].

### Subjects

Participants for Weigh-To-Be were recruited from four clinics of a large collaborating managed-care organization (MCO) by way of fliers, mailings, and physician referral. Members of the MCO interested in the study were invited to call a general phone number to determine eligibility. Eligibility requirements were age ≥ 18 years; not currently pregnant, lactating, or planning to become pregnant in the following 6 months; a BMI ≥ 27 based on self-reported weight and height; and agreement to conditions of participation.

Once recruited, participants were randomized to one of three conditions (a mail-based weight intervention, a telephone-based weight intervention, or a usual care group) by study coordinators, using identification numbers taken in order from the appointment book. Randomization was done in blocks of 15. After randomization, letters were sent to participants to inform them of group status. Weight loss protocols were derived from prior weight loss studies by members of the Weigh-to-Be research team and designed as potentially cost-effective delivery modes for weight loss within an MCO population. Intervention consisted of 10 self-paced modules that promoted behavioral goal setting and self-monitoring, decreases in caloric intake, and increases in physical activity. Intervention content was designed for delivery by mail (written materials and regular mailings from trained staff to monitor treatment progress) or by telephone (same written materials as mail-based intervention, with the substitution of regular telephone contacts with trained staff to monitor treatment progress). Participants assigned to the usual care group were not offered weight intervention beyond that available through the MCO health plan. At the time of the study, the MCO health plan was offering telephone-based weight counseling, clinic-based advice on selecting a weight control program in the community, and clinic-based group programs provided by health plan staff, both for a $25 fee.

Data were collected from 508 men and 1293 women at baseline, and from 295 men and 705 women at 24-month follow-up (56% of baseline sample). Participants with missing data at follow-up were younger [48.4 vs. 52.6 years, *t*(1652) = 7.24, *p *< .0001], less educated [3.2 vs. 3.5 on a 5-point scale, *t*(1798) = 4.92, *p *< .0001], and had a higher average baseline BMI [34.8 vs. 33.7 kg/m^2^, *t*(1638) = -4.06, *p *< .0001]. No significant differences in weight change between participants in the three treatment conditions were observed at two years (mail group: -0.7 kg, telephone group: -1.0 kg, usual care group: -0.6 kg; *p *= .55) [15].

### Measures

#### Body mass index (BMI)

Participant height and weight were measured using a calibrated electronic scale and a wall-mounted ruler. Height was measured at baseline only, and weight was measured at both baseline and 24-month follow-up. BMI was calculated as weight (kilograms) divided by height (meters) squared. A categorical BMI variable was created to illustrate the magnitude of weight-behavior associations at baseline. Cut points (< 30.0: Overweight, 30.0–34.9: Obese Class I, = 35.0: Obese Classes II and III) were based on World Health Organization classification categories of overweight and obesity [16], with collapse of BMI = 35.0-39.9 (Obese Class II) and BMI > 40 (Obese Class III) categories due to smaller numbers of participants meeting these cut points in this sample relative to lower BMI categories, particularly for men (Obese Class II: n = 62 for men; n = 217 for women; Obese Class III: n = 80 at baseline for men; n = 273 for women; see Table 2 for sample sizes for BMI < 30.0 and BMI = 30.0–34.9 categories).

**Table 1:** Characteristics of the study population.

	Men	Women
	N (%) or Mean (SD)	N (%) or Mean (SD)

Total	508 (28%)	1293 (72%)
Mean age (years)	53.7 (0.5)	49.5 (0.3)
Education (some college or greater)	415 (82%)	981 (76%)
Mean BMI (kg/m^2^), baseline	33.6 (0.3)	34.4 (0.2)
Mean BMI change, 24 months	-0.46 (2.35)‡	-0.49 (2.66)‡

Consumption of selected foods and total fat/fiber at baseline (mean frequency/month)		

Hamburgers	4.4 (3.6)	3.3 (2.5)
Beef	4.7 (3.7)	4.1 (3.3)
Fried chicken	2.6 (2.7)	1.9 (2.0)
Hot dogs	2.2 (2.1)	1.6 (1.3)
Eggs	5.1 (3.8)	4.8 (3.7)
Bacon or sausage	3.3 (3.0)	2.7 (2.6)
French fries	3.7 (3.2)	3.0 (2.6)
Total fat	82.3 (28.7)	74.7 (26.0)
Fruit	14.2 (9.8)	16.6 (10.5)
High fiber cereal	9.7 (9.7)	8.4 (9.2)
Total fruit/vegetable/fiber	97.1 (36.0)	97.4 (36.8)

Consumption of selected foods at 24 months (mean frequency/month)		

Hamburgers	3.0 (2.5) ‡	2.4 (1.7)‡
Beef	3.6 (2.7)‡	3.1 (2.4)‡
Fried chicken	2.2 (2.1)*	1.5 (1.2)‡
Hot dogs	1.8 (2.0)	1.5 (1.4)
Eggs	5.3 (4.4)	4.7 (3.7)
Bacon or sausage	2.9 (2.9)	2.3 (2.3)‡
French fries	2.5 (2.0)‡	2.2 (1.9)‡
Total fat	65.5 (24.9)§	59.3 (22.9)§
Fruit	17.7 (9.9)‡	20.1 (10.1)‡
High fiber cereal	12.2 (10.3)*	10.1 (9.7)‡
Total fruit/vegetable/fiber	108.5 (36.4)†	104.5 (35.6)§
Ever dieted to lose weight	360 (71%)	1183 (92%)
Formal weight loss program, past 2 years	72 (14%)	455 (35%)
Own weight loss books	198 (39%)	721 (56%)
Own nutrition books	252 (50%)	766 (59%)
Own low fat cookbooks	303 (57%)	984 (76%)
Subscribe to exercise magazines	37 (7%)	97 (7%)

Note. Significant difference from baseline (*t*-test or Fisher's exact test): *p < .05. †*p *< .01. ‡*p *< .001. §*p *< .0001.

**Table 2:** Mean baseline BMI by weight-related behavior.

	Men			Women		
	Yes	No		Yes	No	

	Mean (SE)	Mean (SE)	*p*	Mean (SE)	Mean (SE)	*p*

Diet program (past 2 years)	35.46 (0.58)	33.30 (0.24)	< .01	35.00 (0.30)	34.12 (0.22)	.02
Ever dieted	34.17 (0.26)	32.22 (0.41)	< .01	34.67 (0.18)	31.80 (0.60)	< .01
Weight loss books at home	34.69 (0.35)	32.92 (0.28)	< .01	35.16 (0.23)	33.51 (0.26)	< .01
Nutrition books at home	33.78 (0.32)	33.43 (0.31)	.43	34.94 (0.23)	33.69 (0.27)	< .01
Diet cookbooks at home	33.56 (0.29)	33.68 (0.35)	.80	34.74 (0.20)	33.43 (0.36)	< .01
Exercise magazine subscription	33.04 (0.82)	33.65 (0.23)	.48	33.03 (0.64)	34.54 (0.18)	.02

Note. *N *= 508 men, 1293 women. All analyses controlled for age and education level.

#### Dietary variables

The Block Screening Questionnaire for Fat [17] was used to assess consumption of commonly eaten high-fat and high-sugar foods. The scale is comprised of 15 items and has been shown to be a valid measure for identifying individuals with high percentages of fat intake [18]. The frequency of consumption of the 15 foods was assessed with the question, "Think about your eating habits over the past year or so. About how often do you eat each of the following foods?" Five response categories were recoded into frequency per month as follows: "less than once per month" = 1; "2–3 times per month" = 2.5; "1–2 times per week" = 6; "3–4 times per week" = 14; "5 times per week" = 20. A composite index of all fat servings, coded as servings per month, was calculated along with individual food item monthly serving frequencies. The scale was administered at baseline and at 24-month follow-up.

The Block Screening Questionnaire for Fruit/Vegetable/Fiber Intake includes 9 items assessing frequency of consumption of commonly eaten high-fiber foods [17]. The measure has acceptable validity for identifying individuals with high percentages of intake of the foods assessed [18]. Participants were asked, "How often do you eat each of the following foods?" Five response categories were recoded to frequency per month as follows: "less than once per week" = 2; "about 1 time per week" = 4; "2–3 times per week" = 10; "4–5 times per week" = 18; "every day" = 30. A composite index of all fruit/vegetable/fiber servings, coded as servings per month, was calculated along with individual food item monthly serving frequencies. This scale was administered at baseline and at 24-month follow-up.

#### Weight-related behaviors

Participants were asked with single-item questions if they had ever dieted, if they had participated in commercial weight-loss programs during the previous two years, and if they owned any weight-loss books, books about nutrition, low-fat cookbooks, or subscribed to exercise magazines. These data were collected at baseline only.

#### Personal characteristics

Participant's age and highest level of educational attainment were measured by self-report at baseline.

### Data analysis

Cross-sectional and longitudinal analyses were conducted using SAS statistical software (SAS Version 8, Cary, North Carolina, 1999). All analyses were stratified by gender. Longitudinal analyses examining relationships between change in weight and change in predictor variables also controlled for baseline weight, baseline predictor values, age and education. Means and frequencies were calculated to describe personal characteristics and weight-related behaviors of men and women in the study population (Table 1). Linear multivariate models were used to examine the relationships between BMI and frequency of consumption of individual food items and food composite scores [see Additional file 1]. Models were run with BMI as a categorical variable. Multivariate linear models with BMI as the dependent variable were next used to estimate average BMI by level of weight-related behavior (Table 2). Lastly, linear multivariate models were used to examine associations of baseline behaviors with 24-month BMI change, and to examine the associations of changes in diet with 24-month BMI change (Table 3); to account for the possibility of differential changes in dietary intake over time due to treatment group assignment, these models were also examined with treatment group assignment included as a covariate. Effect size coefficients (Cohen's *d*) were calculated for all dietary variables to assist in interpretation of the relative contribution of changes in individual foods or composite food indices to BMI change (small = .20, medium = .50, large = .80) [19].

**Table 3:** Associations of changes in individual food intake and physical activity with BMI change to 24 months.

	24-Month BMI Change							
	Men				Women			

Change in Food Intake	*B*	*SE*	*p*	*d*	*B*	*SE*	*p*	*d*

Hamburgers	0.17	0.06	< .01	.36	0.32	0.06	< .01	.37
Beef	0.18	0.05	< .01	.40	0.19	0.05	< .01	.32
Fried chicken	0.04	0.07	.52	.08	-0.02	0.09	.83	.02
Hot dogs	0.15	0.07	.02	.28	0.19	0.09	.04	.16
Cold cuts	0.00	0.03	.97	.00	-0.03	0.03	.28	.09
Salad dressings, mayonnaise	0.09	0.03	< .01	.33	0.06	0.03	.02	.18
Margarine or butter	0.08	0.02	< .01	.43	0.09	0.02	< .01	.42
Eggs	-0.00	0.04	.99	.01	-0.00	0.03	.99	.00
Bacon or sausage	0.06	0.05	.19	.16	0.20	0.06	< .01	.26
Cheese	0.06	0.03	.03	.26	0.05	0.02	.01	.19
Whole milk	-0.02	0.05	.66	.06	0.08	0.05	.11	.12
French fries	0.32	0.07	< .01	.56	0.18	0.06	< .01	.23
Potato chips, corn chips, popcorn	0.10	0.04	< .01	.33	0.05	0.03	.07	.14
Ice cream	0.10	0.04	.01	.30	0.08	0.03	< .01	.20
Doughnuts, pastries, cake, cookies	0.09	0.03	< .01	.35	0.05	0.02	.02	.17
Total fat	0.04	0.01	< .01	.69	0.03	0.01	< .01	.41
Orange juice	-0.02	0.02	.30	.11	0.01	0.01	.29	.08
Fruit, not counting juice	-0.07	0.02	< .01	.52	-0.04	0.01	< .01	.25
Green salad	-0.06	0.02	< .01	.31	-0.06	0.02	< .01	.26
Potatoes	0.04	0.03	.13	.18	0.04	0.02	.10	.13
Beans	-0.03	0.03	.34	.11	-0.02	0.03	.40	.06
Other vegetables	-0.05	0.02	< .01	.31	-0.06	0.01	< .01	.35
High-fiber or bran cereal	-0.05	0.02	< .01	.38	-0.05	0.01	< .01	.29
Dark bread	-0.04	0.02	.02	.29	-0.02	0.01	.11	.12
White bread	0.02	0.02	.31	.12	0.02	0.01	.10	.13
Total fruit/vegetable/fiber	-0.02	0.01	< .01	.51	-0.02	0.00	< .01	.29

Note. *N *= 291 men, 697 women. Analyses controlled for age, education level, baseline BMI (continuous), and baseline food intake (continuous; servings per month). For Cohen's effect size statistic (*d*), small = .20, medium = .50, large = .80.

## Results

### Sample characteristics

Demographic characteristics of the sample are described in Table 1. The study population had a mean age of 51 years. Over two-thirds were clinically obese, mean BMI = 34 kg/m^2^. Most participants had reported some college education. Most also reported a history of dieting. Almost one-third had enrolled in a formal weight-loss program in the previous two years. By 24 months, study participants had lost an average of 0.50 BMI units. Reported consumption of specific foods (e.g., hamburgers, beef, fried chicken, French fries) and total fat intake had decreased significantly over the two year study period, and consumption of some high-fiber foods (e.g., fruit, high fiber cereal) and total fiber intake had increased significantly over the two years.

### Baseline associations between weight control behaviors and BMI

Cross-sectional associations between baseline frequency of consumption of the 24 individual food items or overall fat and fiber and baseline BMI [see Additional file 1] indicate that for men, higher BMI was significantly associated with greater consumption of hamburgers, beef, fried chicken, and white bread, with effects primarily due to higher consumption in men with BMI ≥ 35. For women, higher BMI was significantly associated with greater consumption of hamburgers, beef, fried chicken, hot dogs, eggs, bacon or sausage, and French fries, as well as with overall greater fat intake. Lower BMI was significantly associated with greater consumption of fruit and high-fiber cereal as well as with overall greater fiber intake.

Cross-sectional relationships between BMI and weight-related behaviors are reported in Table 2. Both men and women who reported having ever dieted to lose weight, enrolling in a commercial weight-loss program, or who owned weight-loss books had mean BMIs approximately two units higher than those not reporting those behaviors. Women who owned nutrition books and diet cookbooks had a significantly higher mean BMI than women who did not own those materials. Women who subscribed to exercise magazines had a significantly lower BMI than those who did not.

### Associations of weight control behaviors with 24-month BMI change

Next, relationships between baseline food intake or other weight control behaviors and 24-month BMI change were examined using general linear models that controlled for age, education, and baseline BMI. The only baseline behavior to be significantly associated with BMI change at 24 months was self-reported participation in weight control programs during the previous two years. Those who had reported a history of participation prior to baseline had lost less weight by 24 months (adjusted mean BMI change for men: yes = +0.49 vs. no = -0.62, *p *< .01; for women: yes = -0.05 vs. no = -0.74, *p *< .01).

For the individual food items, several associations between individual food intake at baseline and BMI change at 24 months were observed. Baseline levels of hot dog and French fry consumption were associated with BMI change for men and women, though in opposite directions. For men, greater baseline intake of hot dogs and French fries was associated with better BMI outcomes at 24 months (hot dogs: *B *= -0.19, *p *< .01; French fries: *B *= -0.13, *p *< .05), whereas for women, greater consumption of these foods was associated with poorer BMI outcomes (hot dogs: *B *= 0.18, *p *< .05; French fries: *B *= 0.09, *p *< .05). The following foods were associated with better BMI outcomes at 24 months as well: cold cuts and pastry for men (cold cuts: *B *= -0.05, *p *< .05; pastry: *B *= -0.06, *p *< .01), and ice cream for women (*B *= -0.04, *p *< .05). In addition, greater overall fat intake at baseline was associated with better BMI outcomes for men only (*B *= -0.01, *p *< .05).

Lastly, associations of changes in individual food intake and BMI change between baseline and 24 months are presented in Table 3. Effect sizes were in the medium range for total fat and total fiber indices (*d *= .51–.69 for men, .29–.41 for women), and in the small to medium range for individual foods (*d *= .00–.56 for men, .00–.42 for women). Statistically significant results indicate that changes in fat intake were positively associated with BMI change, and changes in fruit, vegetable, or fiber intake were inversely associated with BMI change. For men, increased hamburger, beef, hot dog, salad dressing, margarine, cheese, French fry, chip, ice cream, pastry and total fat intake over the course of the study were associated with increases in BMI, and increased fruit, green salad, vegetable, high-fiber cereal, dark bread and total fiber intake were associated with decreases in BMI. For women, increased hamburger, beef, hot dog, salad dressing, margarine, bacon, cheese, French fry, ice cream, pastry, and total fat intake over the course of the study were associated with increases in BMI, and increased fruit, green salad, vegetable, high-fiber cereal, and total fiber intake were associated with decreases in BMI. The results for all analyses of 24-month BMI change with baseline food intake or changes in food intake were unaffected by inclusion of treatment group assignment in the models.

## Discussion

A major purpose of the present paper was to determine whether behaviors that are associated with BMI in general population samples are similarly associated with BMI in overweight and obese persons seeking treatment. The answer to this question appears to be yes. In this sample, over two-thirds of participants were clinically obese; about one-third were severely obese. The results of the present investigation show that observations from general population samples that frequency of consumption of specific high-fat foods (particularly meat products) are positively associated with body weight [8,13,20-22] and, to a lesser extent, that intake of fruits and vegetables are inversely associated with body weight [10,23], are generalizable to much higher body weights as well. Likewise, positive changes in dietary intake over the course of the study (i.e., decreases in intake of high-fat foods; increases in intake of fruits, vegetables, or high-fiber foods) were associated with favorable changes in BMI. In other words, obese and very obese treatment may have more difficulty balancing their eating and activity behaviors, but the relationships between specific behaviors and body weight are on the same continuum as the rest of the population.

Results in this population relating to dieting history and behaviors are also strongly similar to those seen in weight-maintaining populations or those with lower BMI [24-26]. Individuals with higher BMI diet more and participate in formal diet programs more often than those with lower BMI. It is likely that those who have engaged in subsequent attempts to reduce BMI do so because they have not been successful in earlier attempts; it is possible either that they have not engaged in weight loss behaviors at the level or duration required to sustain weight loss [27], or that the pattern of repeated weight losses and gains promotes larger weight gains over time [28]. New findings from this research extend the observations about greater global dieting concerns to possession of diet-related books. Those with higher BMI own more books about weight loss, nutrition, and diet cooking. Of some interest is the observation that those who owned subscriptions to magazines about exercise had lower BMI than those who did not, although the percentage of individuals with exercise magazines was small (< 10%).

Interestingly, higher baseline consumption of several high-fat foods (e.g., hot dogs, pastries, French fries, ice cream), as well as total fat for men, was associated with lower 24-month BMI. This pattern was most notable for men, who, relative to women, tend to consume a higher-fat diet in general and who may not value healthy eating habits as strongly [29]. In every case, however, reductions in consumption of these specific foods over time, and overall reductions in fat intake, were associated with better BMI outcomes for both men and women. It is likely that these high-fat foods are readily identified targets for intake modification among high consumers who engage in a weight loss program, thus leading to an association of baseline consumption with BMI reduction over time. Whereas effect size indicators suggest that changes in composite indices of fat or fiber intake were stronger predictors of BMI change that individual foods, changes in intake of some individual foods predicted BMI change as well as composite measures (e.g., French fries or fruit for men; margarine, vegetables, or cereal for women). It is likely that these foods, which may be identified easily as sources of fat or dietary fiber, also became obvious targets for reduced or increased consumption within the context of a dietary change plan, as advocated in the weight loss program in which study participants were enrolled.

It should be noted that in the Weigh-to-Be trial, participation in weight loss activities was high even in the usual care group, such that the three groups (usual care, mail intervention, and phone intervention) did not differ in terms of weight change at the 2-year follow-up [15]. Thus, regardless of treatment group assignment in this trial, those individuals who consumed more of these foods at baseline may have had more options for overall fat reduction at their disposal during the weight loss intervention, thus assisting their weight loss efforts.

This study was not without limitations. Dietary intake was assessed by screening instruments selected for the main study that may be limited in their ability to capture complete, well-differentiated dietary information. Attrition from the study over the two years was relatively high; however, the factors on which study completers differed from dropouts (i.e., age, education, BMI) were included in the statistical models, and adjustment for attrition by imputation of missing values using the last observation carried forward [30] did not change the findings presented here. Lastly, BMI is not a direct measure of adiposity; as such, BMI may misclassify overweight or obese status depending on age, gender, ethnicity, athleticism, or redistribution of proportions of fat mass versus lean mass by resistance training [31,32].

## Conclusion

Earlier work in this domain has suggested that reported changes in specific food consumption account for greater proportions of variance in weight change than reported changes in macronutrient consumption [7]. Whereas changes in intake of total fat and fiber-rich foods were predictive of weight change over time, individual foods were significantly predictive as well. Taken with the current findings, these data are consistent with the argument that a public health intervention focus on reducing consumption of specific foods (e.g., hamburgers, French fries, or processed meats) and increasing consumption of other specific foods such as fruits, vegetables, or other high-fiber foods (e.g., bran cereals) might be useful, rather than presenting broad recommendations about foods with different macronutrient composition that the general public may have difficulty translating into food-specific behavioral choices.

## Competing interests

The author(s) declare that they have no competing interests.

## Authors' contributions

JL and JU performed statistical analyses and drafted the manuscript. RJ, NS, NP, and RB developed the study, participated in its design and coordination, and reviewed manuscript drafts. All authors read and approved the final manuscript.

## Supplementary Material

Additional file 1 Associations of BMI categories with consumption of individual foods at baseline. The data provided represent cross-sectional associations between baseline frequency of consumption of the 24 individual food items or overall fat and fiber and baseline BMI for men and women. Click here for file
