# Supplementary material for: Specific food intake, fat and fiber intake, and behavioral correlates of BMI among overweight and obese members of a managed care organization
Source: Int J Behav Nutr Phys Act. 2006 Nov 26;3:42. doi: 10.1186/1479-5868-3-42 (PMC1684256; doi:10.1186/1479-5868-3-42)
Supplement: Additional file 1 — Associations of BMI categories with consumption of individual foods at baseline. The data provided represent cross-sectional associations between baseline frequency of consumption of the 24 individual food items or overall fat and fiber and baseline BMI for men and women. [file 1479-5868-3-42-S1.doc]

**Additional file 1.** Associations of BMI categories with consumption of individual foods at baseline.

|  | BMI < 30 | BMI=30-34.9 | BMI > 35 |  |
| --- | --- | --- | --- | --- |
| Food Consumption (per month) | Mean (SE) | Mean (SE) | Mean (SE) | *B* |
| Men | n=118 (23%) | n=246 (48%) | n=143 (28%) |  |
| Hamburgers | 4.0 (0.3) | 4.0 (0.2) | 5.4 (0.3) | 0.16‡ |
| Beef | 4.5 (0.3) | 4.4 (0.2) | 5.5 (0.3) | 0.11‡ |
| Fried chicken | 2.0 (0.3) | 2.6 (0.2) | 2.8 (0.2) | 0.05* |
| Hot dogs | 2.2 (0.2) | 2.2 (0.1) | 2.2 (0.2) | 0.03 |
| Cold cuts | 6.1 (0.5) | 6.2 (0.3) | 5.2 (0.5) | -0.02 |
| Salad dressings, mayonnaise | 7.0 (0.5) | 6.5 (0.3) | 6.2 (0.5) | 0.01 |
| Margarine or butter | 15.1 (0.6) | 13.9 (0.4) | 14.4 (0.6) | 0.03 |
| Eggs | 5.0 (0.4) | 4.9 (0.2) | 5.4 (0.3) | 0.06 |
| Bacon or sausage | 3.3 (0.3) | 3.2 (0.2) | 3.4 (0.3) | 0.02 |
| Cheese | 8.8 (0.6) | 8.4 (0.4) | 8.8 (0.5) | 0.02 |
| Whole milk | 3.0 (0.4) | 2.1 (0.3) | 2.6 (0.4) | -0.01 |
| French fries | 3.6 (0.3) | 3.5 (0.2) | 4.2 (0.3) | 0.04 |
| Potato chips, corn chips, popcorn | 7.0 (0.5) | 5.9 (0.3) | 6.9 (0.4) | 0.05 |
| Ice cream | 5.4 (0.4) | 4.6 (0.3) | 4.5 (0.4) | -0.02 |
| Doughnuts, pastries, cake, cookies | 8.4 (0.6) | 7.4 (0.4) | 7.3 (0.5) | -0.06 |
| Total fat | 85.6 (2.7) | 79.4 (1.8) | 84.6 (2.4) | 0.47 |
| Orange juice | 12.9 (0.9) | 10.8 (0.6) | 10.2 (0.8) | -0.16 |
|  | BMI < 30 | BMI=30-34.9 | BMI > 35 |  |
| Food Consumption (per month) | Mean (SE) | Mean (SE) | Mean (SE) | *B* |
| Men | n=118 (23%) | n=246 (48%) | n=143 (28%) |  |
| Fruit, not counting juice | 15.0 (0.9) | 14.5 (0.6) | 13.2 (0.8) | -0.07 |
| Green salad | 10.8 (0.6) | 9.3 (0.4) | 8.8 (0.5) | -0.06 |
| Potatoes | 10.3 (0.6) | 9.1 (0.4) | 9.9 (0.5) | 0.03 |
| Beans | 5.0 (0.4) | 4.6 (0.3) | 4.3 (0.4) | -0.03 |
| Other vegetables | 17.1 (0.8) | 16.8 (0.6) | 16.5 (0.8) | -0.07 |
| High-fiber or bran cereal | 10.6 (0.9) | 9.7 (0.6) | 9.0 (0.8) | -0.13 |
| Dark bread | 13.0 (0.9) | 12.6 (0.6) | 11.8 (0.8) | -0.13 |
| White bread | 8.6 (0.7) | 9.3 (0.6) | 10.5 (0.7) | 0.16* |
| Total fruit/vegetable/fiber | 103.1 (3.1) | 96.4 (2.1) | 93.6 (2.8) | -0.47 |
| Women | n=365 (28%) | n=437 (34%) | n=490 (38%) |  |
| Hamburgers | 2.9 (0.1) | 3.2 (0.1) | 3.7 (0.1) | 0.06‡ |
| Beef | 3.8 (0.2) | 4.1 (0.2) | 4.3 (0.2) | 0.03* |
| Fried chicken | 1.8 (0.1) | 1.8 (0.1) | 2.1 (0.1) | 0.03‡ |
| Hot dogs | 1.5 (0.1) | 1.7 (0.1) | 1.7 (0.1) | 0.01* |
| Cold cuts | 3.9 (0.2) | 5.2 (0.2) | 4.8 (0.2) | 0.04 |
| Salad dressings, mayonnaise | 5.9 (0.3) | 6.0 (0.3) | 6.2 (0.2) | 0.02 |
| Margarine or butter | 13.9 (0.3) | 14.5 (0.3) | 14.4 (0.3) | 0.04 |
| Eggs | 4.5 (0.2) | 4.6 (0.2) | 5.2 (0.2) | 0.05† |
| Bacon or sausage | 2.3 (0.1) | 2.5 (0.1) | 3.1 (0.1) | 0.05‡ |
|  | BMI < 30 | BMI=30-34.9 | BMI > 35 |  |
| Food Consumption (per month) | Mean (SE) | Mean (SE) | Mean (SE) | *B* |
| Women | n=365 (28%) | n=437 (34%) | n=490 (38%) |  |
| Cheese | 8.8 (0.3) | 8.6 (0.3) | 8.8 (0.3) | 0.02 |
| Whole milk | 1.5 (0.1) | 1.4 (0.1) | 1.6 (0.1) | 0.01 |
| French fries | 2.6 (0.1) | 2.9 (0.1) | 3.3 (0.1) | 0.05‡ |
| Potato chips, corn chips, popcorn | 5.4 (0.3) | 5.7 (0.2) | 6.0 (0.2) | 0.04 |
| Ice cream | 4.6 (0.3) | 4.8 (0.2) | 4.7 (0.2) | 0.01 |
| Doughnuts, pastries, cake, cookies | 8.2 (0.3) | 7.7 (0.3) | 7.7 (0.3) | -0.03 |
| Total fat | 71.3 (1.4) | 74.6 (1.2) | 77.4 (1.2) | 0.44‡ |
| Orange juice | 10.0 (0.5) | 9.4 (0.5) | 10.1 (0.4) | -0.01 |
| Fruit, not counting juice | 17.5 (0.5) | 17.1 (0.5) | 15.5 (0.4) | -0.12† |
| Green salad | 9.9 (0.4) | 10.8 (0.3) | 9.5 (0.3) | -0.05 |
| Potatoes | 8.0 (0.3) | 8.5 (0.3) | 8.8 (0.3) | 0.05 |
| Beans | 4.3 (0.2) | 4.0 (0.2) | 3.9 (0.2) | -0.02 |
| Other vegetables | 18.5 (0.5) | 18.6 (0.5) | 18.0 (0.4) | -0.04 |
| High-fiber or bran cereal | 9.9 (0.5) | 8.7 (0.4) | 7.1 (0.4) | -0.16‡ |
| Dark bread | 12.0 (0.5) | 11.8 (0.5) | 11.4 (0.4) | -0.00 |
| White bread | 9.5 (0.5) | 10.5 (0.4) | 10.4 (0.4) | 0.04 |
| Total fruit/vegetable/fiber | 99.4 (1.8) | 98.9 (1.6) | 94.5 (1.5) | -0.30* |

Note: All analyses controlled for age and education level. Unstandardized regression coefficients (*B*) are based on linear regression analyses of food variables in frequency of consumption per month with BMI as a continuous variable.

**p*<.05. †*p*<.01. ‡*p*<.001.
